# Transaxial or short axis right ventricular volume measurements - which method correlates more closely with main pulmonary artery flow values in children aged 9 years?

**DOI:** 10.1186/1532-429X-15-S1-T10

**Published:** 2013-01-30

**Authors:** Jennifer A Bryant, Keith Godfrey, Mark A Hanson, Lucy Davies, Alison Fletcher, Charles Peebles

**Affiliations:** 1 NIHR Southampton Biomedical Research Centre, University of Southampton and University Hospital Southampton NHS Foundation Trust, Southampton, UK; 2 Cardiothoracic Radiology, University Hospital Southampton NHS Foundation Trust, Southampton, UK; 3 Institute of Developmental Sciences, University of Southampton, Southampton, UK; 4 MRC Lifecourse Epidemiology Unit, Southampton, UK

## Background

In adults right ventricular (RV) measurements from datasets acquired in the transaxial orientation have been found to be more accurate and reproducible than in the short axis plane. Cardiovascular magnetic resonance imaging (CMRI) in children is prone to image misregistration due to less consistent breath-holding which may be more problematic when imaging in the transaxial plane. This study aimed to determine the most accurate method for RV volume evaluation in children.

## Methods

216 healthy children aged 9 years underwent CMRI as part of a study of developmental influences on cardiovascular structure and function. Contiguous steady state free precession breath-hold cine images were obtained to cover the RV in both short axis and transaxial planes. RV stroke volumes (SV) were compared with flow data derived from phase contrast velocity flow mapping sequences through the main pulmonary artery (MPA).

## Results

Full data was obtained in 189 children. Mean (SD) short axis RVSV and transaxial RVSV were 48.1ml (10.16) and 51.1ml (10.23) respectively.

There was a good correlation between short axis RVSV and MPA Flow volumes (r=0.64, p<0.0001, n=189), but a less strong correlation between transaxial RVSV and MPA flow volumes (r=0.53, p<0.0001, n=189). Using a Bland Altman analysis the mean difference between short axis RVSV and MPA flow volumes was 12ml (95%CI 10.8 to 13.2). Mean difference between transaxial RVSV and MPA Flow volumes was 8.72ml (95%CI 6.73 to 9.82).

Measured transaxial RVSVs was on average 3.76ml (95%CI -5.1 to -2.3ml) greater than short axis RVSV. There was a good correlation between short axis and transaxial RVSVs (r=0.63, p<0.0001, n=216).

## Conclusions

In children RVSV analysis in the short axis plane correlates more closely with MPA flow derived SVs than those from the transaxial plane. This is likely to be due to greater misregistration error in the transaxial plane. One limitation of the study is that the MPA flow was obtained immediately after the short axis stack, whereas the transaxial stack was acquired much later in the protocol. RV and LV measurements can be obtained from a single short axis stack, reducing the total imaging time for children of this age for research studies.

## Funding

This work was supported by funding from the British Heart Foundation and the National Institute for Health Research (NIHR Southampton Biomedical Research Centre).

